# Impact of sarcopenia in patients with advanced non–small cell lung cancer treated with PD-1 inhibitors: A preliminary retrospective study

**DOI:** 10.1038/s41598-019-39120-6

**Published:** 2019-02-21

**Authors:** Takayuki Shiroyama, Izumi Nagatomo, Shohei Koyama, Haruhiko Hirata, Sumiyuki Nishida, Kotaro Miyake, Kiyoharu Fukushima, Yuya Shirai, Yuichi Mitsui, So Takata, Kentaro Masuhiro, Moto Yaga, Kota Iwahori, Yoshito Takeda, Hiroshi Kida, Atsushi Kumanogoh

**Affiliations:** 1 Department of Respiratory Medicine and Clinical Immunology, Graduate School of Medicine, Osaka University, Osaka, Japan; 2 Laboratory of Immunopathology, WPI Immunology Frontier Research Center, Osaka University, Suita, Osaka Japan; 3 Integrated Frontier Research for Medical Science Division, Institute for Open and Transdisciplinary Research Initiatives, Osaka University, Suita, Osaka Japan

## Abstract

The aim of this study was to investigate the clinical impact of sarcopenia on the efficacy of programmed death (PD)-1 inhibitors. We retrospectively reviewed the medical records of all patients treated with nivolumab or pembrolizumab between January 2016 and September 2018 for previously treated advanced non–small cell lung cancer (NSCLC). The cross-sectional area of the psoas muscle at the level of the third lumbar vertebra on baseline computed tomography was assessed to calculate the psoas muscle index (PMI). Sarcopenia was defined based on PMI cut-off values for Asian adults (6.36 cm^2^/m^2^ for males and 3.92 cm^2^/m^2^ for females). A total of 42 patients were analysed. The prevalence of sarcopenia was 52.4%. Sarcopenia was significantly associated with poorer progression-free survival (PFS) (median, 2.1 vs. 6.8 months, *p* = 0.004). Compared to patients with sarcopenia, those without sarcopenia had a higher overall response rate (40.0% vs. 9.1%, *p* = 0.025) and 1-year PFS rate (38.1% vs. 10.1%). In conclusion, sarcopenia at baseline as determined using computed tomography is a significant predictor of worse outcome in patients with advanced NSCLC receiving PD-1 blockade. Screening for sarcopenia may help identify patients more likely to achieve a long-term response in routine clinical practice.

## Introduction

Programmed death (PD)-1 inhibitors such as nivolumab and pembrolizumab demonstrated promising efficacy for treating many cancers, including lung cancer^1–3^. These drugs make it possible to achieve durable responses that are superior to results with conventional cytotoxic anticancer drugs. According to the 3-year update of the CheckMate 017 and 057 trials^4^, 1- and 3-year estimated progression-free survival (PFS) rates were higher in patients treated with nivolumab (20% and 10%, respectively) versus docetaxel (9% and <1%, respectively). It is not satisfactory that long-term response is currently limited to a subset of patients. Various predictive biomarkers for immune checkpoint inhibitors such as PD-ligand 1 (PD-L1) and tumour mutation burden have been reported^5,6^. However, these predictive markers are incomplete, at least when used alone. Additional predictive, easily measured biomarkers that can complement the currently available biomarkers are needed to identify patients who will achieve a durable response to PD-1 inhibitor therapy.

Skeletal muscle loss, referred to as sarcopenia, is a condition characterised by loss of skeletal muscle mass and function. It is a well-established prognostic factor associated with poor outcomes for many cancers^7–9^. Tumour progression is caused by the imbalance between the host and the tumour; it depends on the ability of the host to mount a protective antitumour immune response^10^. Immune checkpoint inhibitors enhance antitumour immunity by blocking the negative regulator of T cell activation, thus promoting the host immune system’s ability to attack cancer cells. Accordingly, the efficacy of immune checkpoint inhibitors is thought to be heavily dependent on the host’s immune system; body composition is strongly associated with the host’s immune system. However, little is known about the clinical impact of skeletal muscle loss in patients with lung cancer treated with PD-1 inhibitors. Computed tomography (CT) is the gold standard method for analysing skeletal muscle mass because it can be performed as part of daily clinical practice. Measurements of the cross-sectional area of skeletal muscles on abdominal CT at the level of the third lumbar vertebra (L3) are widely used to evaluate sarcopenia^7,8,11–13^. The psoas muscle index (PMI) at the L3 level has been used as a surrogate marker of skeletal muscle mass^14–16^.

We conducted a preliminary retrospective study using PMI evaluation to investigate the relationship between sarcopenia and treatment outcomes, including long-term response to PD-1 inhibitors, in patients with previously treated advanced non–small cell lung cancer (NSCLC).

## Results

### Patients

A total of 42 patients with previously treated advanced NSCLC were included in this analysis. Twenty-two patients (52.4%) was identified with sarcopenia based on PMI cut-offs for Asian adults^14^. The baseline clinicopathological characteristics of the patients by sarcopenia status are summarised in Table 1. Male patients were more likely to have sarcopenia than female patients (*p* = 0.055). There were no significant differences in Eastern Cooperative Oncology Group (ECOG) performance status (PS), histology, smoking status, number of prior therapies, and body mass index (BMI) between patients with and without sarcopenia.

**Table 1:** Baseline patient characteristics by sarcopenia status.

	No sarcopenia (n = 20)	Sarcopenia (n = 22)	*p*-value
Age, median (range), years	69 (37–78)	72 (51–87)	0.11
Sex, n (%)			0.055
Male	9 (45.0%)	17 (77.3%)	
Female	11 (55.0%)	5 (22.7%)	
ECOG PS, n (%)			0.13
0–1	18 (90.0%)	15 (68.2%)	
2–3	2 (10.0%)	7 (31.8%)	
Histology, n (%)			0.49
Squamous	5 (25.0%)	7 (31.8%)	
Non-squamous	15 (75.0%)	15 (68.2%)	
Smoking status, n (%)			0.75
Never smoker	8 (40.0%)	7 (31.8%)	
Current or former smoker	12 (60.0%)	15 (68.2%)	
No. of prior therapies, n (%)			0.54
1	12 (60.0%)	10 (45.5%)	
≥2	8 (40.0%)	12 (54.5%)	
Treatment			0.69
Nivolumab	16 (80.0%)	19 (86.4%)	
Pembrolizumab	4 (20.0%)	3 (13.6%)	
Body mass index, kg/m^2^			
Male	22.6 (17.0–30.5)	20.3 (13.7–27.1)	0.25
Female	21.9 (15.1–27.8)	18.2 (15.3–25.6)	0.74
Psoas muscle index, cm^2^/m^2^			
Male	7.49 (6.36–8.26)	4.50 (3.44–6.23)	<0.001
Female	5.08 (4.31–6.70)	3.37 (2.33–3.77)	<0.001

Abbreviation: ECOG PS, Eastern Cooperative Oncology Group performance status.

### Survival

**Figure 1:**
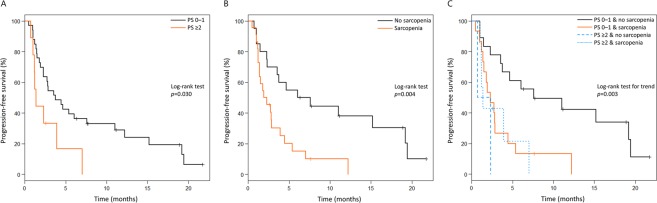
Kaplan-Meier curves of progression-free survival by (**A**) ECOG performance status, (**B**) sarcopenia status, and (**C**) ECOG performance status and sarcopenia status.

The median PFS in the entire cohort was 2.8 months (95% confidence interval: 2.3–7.0 months). Patients with poor PS experienced significantly worse PFS than those with good PS (1.4 vs. 3.8 months, respectively, *p* = 0.030) (Fig. 1A). Patients with sarcopenia experienced significantly worse PFS than those without sarcopenia (2.1 vs. 6.8 months, respectively, *p* = 0.004) (Fig. 1B). In addition, among patients with good PS, sarcopenia was associated with significantly worse PFS than no sarcopenia (2.3 vs. 7.6 months, respectively, *p* = 0.004). On the other hand, among patients with poor PS, there was no significant difference in PFS between patients accompanied by sarcopenia and those without (1.4 vs. 1.5 months, respectively, *p* = 0.30). There was a statistically significant difference in the four groups based on PS and sarcopenia status (log-rank test for trend, *p* = 0.003) (Fig. 1C). By contrast, there were no significant difference in PFS between underweight (BMI < 18.5 kg/m^2^), normal weight (BMI, 18.5–25.0 kg/m^2^), and overweight (BMI > 25.0 kg/m^2^) patients (log-rank test for trend, *p* = 0.1). In the univariate analysis, factors significantly associated with poor PFS included male sex, ECOG PS ≥2, and sarcopenia (Table 2). The hazard ratio of sarcopenia adjusted by sex and PS for PFS was 2.18 (95% confidence interval: 0.92–5.17, p = 0.077). Patients with sarcopenia had a lower 1-year PFS rate than those without (10.1% vs. 38.1%) (Table 3).

**Table 2:** Factors associated with progression-free survival (PFS) in univariate analysis.

	HR (95% CI)	*p*-value
Age		
<70 years	Reference	
≥70 years	1.09 (0.55–2.16)	0.81
Sex		
Female	Reference	
Male	2.54 (1.21–5.33)	0.014
ECOG PS		
0–1	Reference	
≥2	2.44 (1.07–5.59)	0.030
Smoking history		
Never smoker	Reference	
Current or former smoker	1.54 (0.75–3.16)	0.24
Histology		
Non-squamous	Reference	
Squamous	1.32 (0.63–2.76)	0.47
Sarcopenia		
No	Reference	
Yes	2.83 (1.35–5.95)	0.004

Abbreviations: HR, hazard ratio; CI, confidence interval; ECOG PS, Eastern Cooperative Oncology Group performance status.

**Table 3:** Treatment outcome by sarcopenia status.

	No sarcopenia (n = 20)	Sarcopenia (n = 22)	*p*-value
Number of doses, median (range)	11 (1–44)	3.5 (1–26)	0.021
Response rate (95% CI)	40.0% (19.1–63.9%)	9.1% (1.1–29.2%)	0.025
Disease control rate (95% CI)	65.0% (40.8–84.6%)	34.8% (17.2–59.3%)	0.12
1-year PFS (95% CI)	38.1% (21.3–68.1%)	10.1% (2.7–37.3%)	—

Abbreviations: CI, confidence interval; PFS, progression-free survival.

### Response

**Figure 2:**
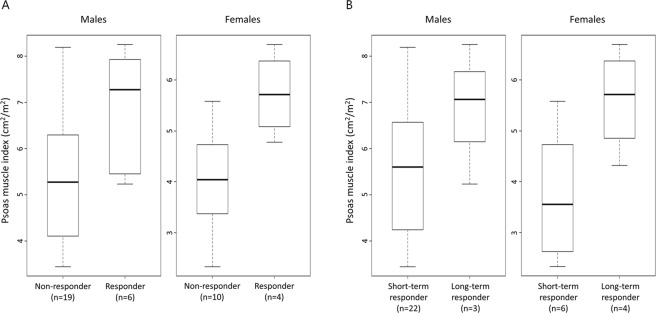
Box plots representing the relationship between treatment outcome and psoas muscle index by sex. (**A**) Best overall response (responders were defined as having complete response or partial response). Three patients who were not evaluable for response were excluded from the analysis. (**B**) Long-term response (long-term responders were defined as having >12 months of tumour remission). Seven patients with follow-up of less than 12 months and no disease progression were excluded from the analysis.

The overall response rate in the entire cohort was 23.8%. Patients with sarcopenia had a worse response rate than patients without sarcopenia (9.1% vs. 40.0%, *p* = 0.025) (Table 3). Long-term response, defined as >12 months of tumour remission, was observed in 7 patients (16.7%). Regarding clinical features correlated with long-term response, there were no significant differences in median age, sex, ECOG PS, smoking history, and histology between patients who achieved long-term response and those who did not. Patients without sarcopenia achieved long-term response than those with sarcopenia (30.0% vs. 4.5%, *p* = 0.041). The distribution of PMI at baseline by treatment response is shown in Fig. 2. PMI was higher in patients who obtained partial response than in those who did not (7.28 vs. 5.28 cm^2^/m^2^ for males, *p* = 0.043, and 5.71 vs. 4.04 cm^2^/m^2^ for females *p* = 0.014) (Fig. 2A). Median PMI was higher in patients who obtained long-term response than in those who did not, but these differences were not statistically significant (7.07 vs. 5.60 cm^2^/m^2^ for males, *p* = 0.24, and 5.71 vs. 3.56 cm^2^/m^2^ for females, *p* = 0.067) (Fig. 2B).

## Discussion

This study investigated the impact of sarcopenia on the efficacy of PD-1 inhibitor therapy in clinical practice. We found that patients without sarcopenia at baseline had better PFS and were more likely to achieve a more favourable best overall response and long-term response than patients with NSCLC previously treated with nivolumab or pembrolizumab. Our results suggest that baseline skeletal muscle mass has a considerable impact on the efficacy of PD-1 inhibitors and skeletal muscle loss might be a useful predictive marker for treatment outcomes.

Although there are various predictive factors for the efficacy of immune checkpoint inhibitors in clinical practice, such as neutrophil-to-lymphocyte ratio, lactate dehydrogenase, and liver metastasis^6,17–20^, limited data from patients with melanoma treated with PD-1 or cytotoxic T-lymphocyte–associated protein (CTLA)-4 inhibitors indicate that skeletal muscle loss is associated with worse overall survival and higher frequency of toxicity^21,22^. On the other hand, the relationship between sarcopenia and PD-1 inhibitor treatment efficacy has not yet been well evaluated in patients with lung cancer. To the best of our knowledge, this is the first study to investigate the association between sarcopenia and the efficacy of PD-1 blockade and long-term response in patients with previously treated NSCLC. In our cohort, the prevalence of sarcopenia was approximately 50%. Although the prevalence of sarcopenia varies by the primary site of cancer, the prevalence of sarcopenia in our study are generally consistent with rates from earlier reports about lung cancer^15,23,24^. In this study, males had a higher prevalence of sarcopenia than females, which might have led to worse PFS among males. There was no significant difference in ECOG PS between males and females (*p* = 0.71). Regarding differences in the prevalence of sarcopenia by sex, mixed results have been reported in patients with cancer. Some studies reported a higher prevalence of sarcopenia in males than females^7,15,24^. Other studies found that sarcopenia was more prevalent in females^21,25^. It is difficult to accurately interpret sex-based differences because other factors such as ethnicity and genetic predisposition may influence the prevalence of sarcopenia. We found poor PS and sarcopenia were related to poor PFS, but these factors were considered not to be independent of each other. In fact, patients with poor PS had a non-significant tendency to have less skeletal muscle mass than those with good PS, regardless of sex (Supplementary Fig. S1). Among patients with good PS, there was a significant difference in PFS by sarcopenia status. Notably, even among patients with good PS, once sarcopenia is present, they had poorer treatment outcomes, similar to those of patients with poor PS. Furthermore, whereas the 1-year PFS rate was reported to be 20% based on updated data from the CheckMate 017 and 057 trials^4^, patients without sarcopenia in our study achieved a 1-year PFS rate of approximately 40%. Therefore, the impact of sarcopenia should not be ignored in routine practice. Sufficient baseline skeletal muscle mass might be critical for a durable response with PD-1 inhibitors.

There are several possible explanations for the association between sarcopenia and worse outcome with anti-PD-1 treatment. Chronic inflammation, a major contributor to sarcopenia, results in tumour cell immune escape through mechanisms such as T cell exhaustion^26^. Several biomarkers potentially involved in the development of sarcopenia such as tissue growth factor (TGF)-β and interleukin (IL)-6 are considered as factors that might impair tumour response to immune checkpoint inhibitors. It has been reported that TGF-β attenuates tumour response to PD-L1 blockade by restricting T cell infiltration^27^, and combined blockade of IL-6 and PD-1/PD-L1 signalling exerts synergistic antitumour effects^28^. The presence of myokines and PPAR-gamma coactivator (PGC)-1α can also contribute to the explanation of our results. Myokines, factors produced and secreted by skeletal muscle, and PGC-1α, a key exercise factor in muscle, have systemic effects on antitumour immune response^29–32^. Therefore, we speculate that the decrease in myokines and PGC-1α due to skeletal muscle loss may lead to a poor response to PD-1 inhibitors.

Sarcopenia and cancer cachexia are multifactorial syndromes characterised by on-going skeletal muscle wasting frequently observed in patients with advanced cancer^33^. They result in serious clinical outcomes such as poor quality of life, treatment intolerance, and survival. Although there are currently no effective pharmacological treatments for cancer cachexia, except for non-pharmacological treatments such as nutritional therapy and exercise training^34^, several randomised controlled trials recently demonstrated new potential treatments for cancer cachexia such as anamorelin (a ghrelin receptor agonist) and enobosarm (a selective androgen receptor modulator), which increased skeletal muscle mass in patients with advanced NSCLC^35–38^. Such treatment might be important going forward, especially in patients planning to receive anti–PD-1 therapy. Whereas currently available biomarkers such as PD-L1 expression and tumour mutation burden are useful for identifying a subset of patients likely to benefit from immune checkpoint inhibitors, early recognition and treatment of sarcopenia have the potential to bring additional benefits to all patients scheduled to receive immune checkpoint inhibitors. We believe that the prevention of skeletal muscle loss and the development of predictive biomarkers are both important to gaining the greatest benefit from immune checkpoint inhibitors in the future.

This study has several limitations. First, our study was retrospective and had a small sample size, precluding definite conclusions. Second, patients were excluded from our analysis if abdominal CT scan was not performed at baseline, which may lead to selection bias. Finally, because of the lack of information regarding PD-L1 expression and other laboratory findings, we were unable to include these variables in our analyses. Only a small number of patients in this study underwent PD-L1 testing (Supplementary Fig. S2) because PD-L1 status was not routinely tested in patients with NSCLC outside of clinical trials at the time nivolumab was approved in Japan. Our findings should be regarded as hypothesis-generating for future studies.

In conclusion, we found that sarcopenia at baseline is associated with significantly poorer PD-1 inhibitor treatment outcomes in patients with previously treated advanced NSCLC. Screening for sarcopenia can help identify patients likely to achieve a long-term response in clinical practice. Further prospective studies are warranted to validate our findings.

## Materials and Methods

### Patient eligibility

We included all patients with previously treated advanced NSCLC who initiated nivolumab (3 mg/kg intravenously every two weeks) or pembrolizumab treatment (200 mg/body intravenously every three weeks) at our institution between January 1, 2016 and September 30, 2018. Patients were excluded from our analysis if they received nivolumab or pembrolizumab as part of a clinical trial or received any treatment concurrently with other anticancer therapies or did not undergo abdominal CT within 3 months of starting treatment. This study was approved by the Ethics Review Board of our institution. The research was conducted in accordance with the 1964 Declaration of Helsinki and amendments. This research was defined as a study without human samples by the Japanese guidelines presented by the Ministry of Health, Labour and Welfare. Therefore, written informed consent was not required, and we used our institutional official website as an opt-out method.

### Skeletal muscle assessment

The cross-sectional area of the psoas muscle at the caudal end of the L3 level was measured using SYNAPSE VINCENT software (Fujifilm Medical, Tokyo, Japan) before initiating treatment. L3 psoas muscle cross-sectional area was identified and quantified using Hounsfield unit thresholds (−29 to +150)^39^. The total bilateral psoas area at the L3 level was normalised for height using the following equation:

$${\rm{PMI}}({{\rm{cm}}}^{2}/{{\rm{m}}}^{2})={\rm{cross}}-{\rm{sectional}}\,{\rm{area}}\,{\rm{of}}\,{\rm{both}}\,{\rm{psoas}}\,{\rm{muscles}}({{\rm{cm}}}^{2})/{{\rm{height}}}^{2}\,({{\rm{m}}}^{2}).$$

The PMI cut-off values for sarcopenia in our study were 6.36 (cm^2^/m^2^) for males and 3.92 (cm^2^/m^2^) for females based on a previous report defining sarcopenia in Asian adults^14^. The definition of sarcopenia was based only on skeletal muscle mass in the present study.

### Data collection

We retrospectively reviewed medical records. We collected data on patient demographics, ECOG PS, smoking history, histology, previous treatments, and BMI at baseline. The response to nivolumab or pembrolizumab was determined based on Response Evaluation Criteria in Solid Tumors criteria, version 1.1^40^. Dates of progression, death, or last follow-up were specified. The cut-off date for data collection was November 30, 2018.

### Statistical analyses

Survival curves were analysed using the Kaplan-Meier method. Differences between groups were compared using the log-rank test. Univariate analysis was performed using Cox proportional hazards and logistic regression models. Continuous variables were analysed using the Mann-Whitney U test, while categorical variables were analysed using Fisher’s exact test. All *p*-values were two-sided, and a *p*-value < 0.05 was considered statistically significant. All statistical analyses were conducted using R, version 3.5.1.

## Supplementary information

Supplementary Figure S1, S2

## Data Availability

All data generated or analysed in this study are included in this published article.
