# Supplementary material for: Impact of sarcopenia in patients with advanced non–small cell lung cancer treated with PD-1 inhibitors: A preliminary retrospective study
Source: Sci Rep. 2019 Feb 21;9:2447. doi: 10.1038/s41598-019-39120-6 (PMC6385253; doi:10.1038/s41598-019-39120-6)

SUPPLEMENTARY INFORMATION

Impact of sarcopenia in patients with advanced non–small cell lung cancer treated with PD-1 inhibitors: A preliminary retrospective study

Takayuki Shiroyama*, Izumi Nagatomo, Shohei Koyama, Haruhiko Hirata, Sumiyuki Nishida, Kotaro Miyake, Kiyoharu Fukushima, Yuya Shirai, Yuichi Mitsui, So Takata, Kentaro Masuhiro, Moto Yaga, Kota Iwahori, Yoshito Takeda, Hiroshi Kida & Atsushi Kumanogoh.

Department of Respiratory Medicine and Clinical Immunology, Graduate School of Medicine, Osaka University, Osaka, Japan

Corresponding author:

Takayuki Shiroyama, MD; E-mail: takayuki.s12@hotmail.co.jp

Supplementary Figure S1.

Figure S1. Box plots representing the relationship between ECOG performance status and psoas muscle index by sex. One male patient had an ECOG PS of 3.

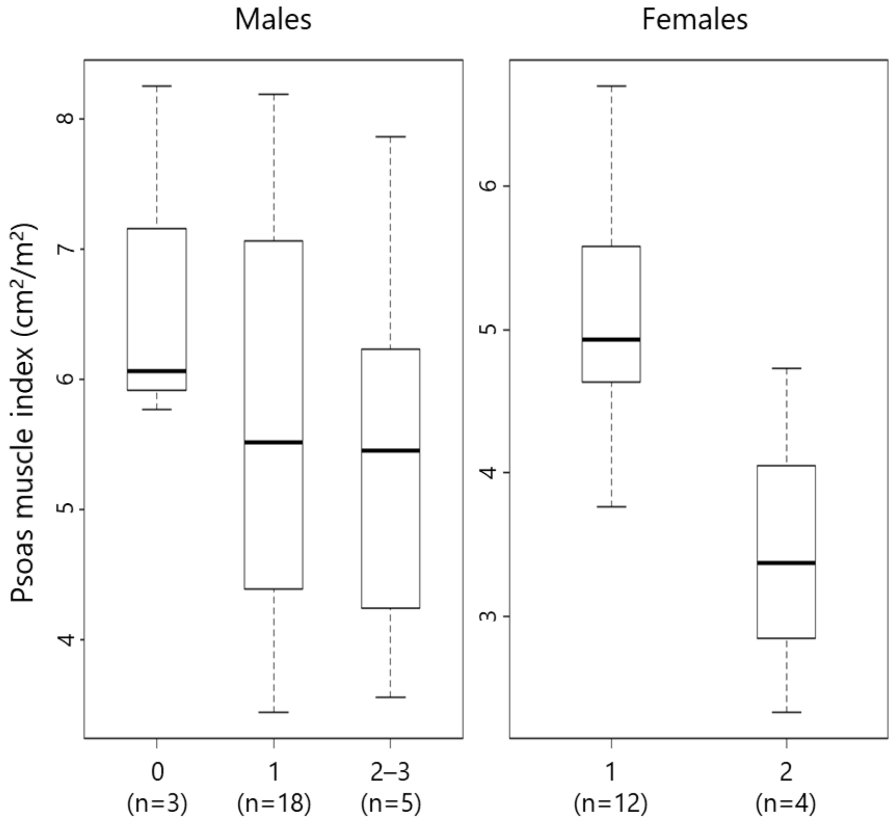

Supplementary Figure S2.

Figure S2. Kaplan-Meier curves of progression-free survival by PD-L1 expression status. PD-L1 expression was tested in 18 patients (42.9%) in this study.

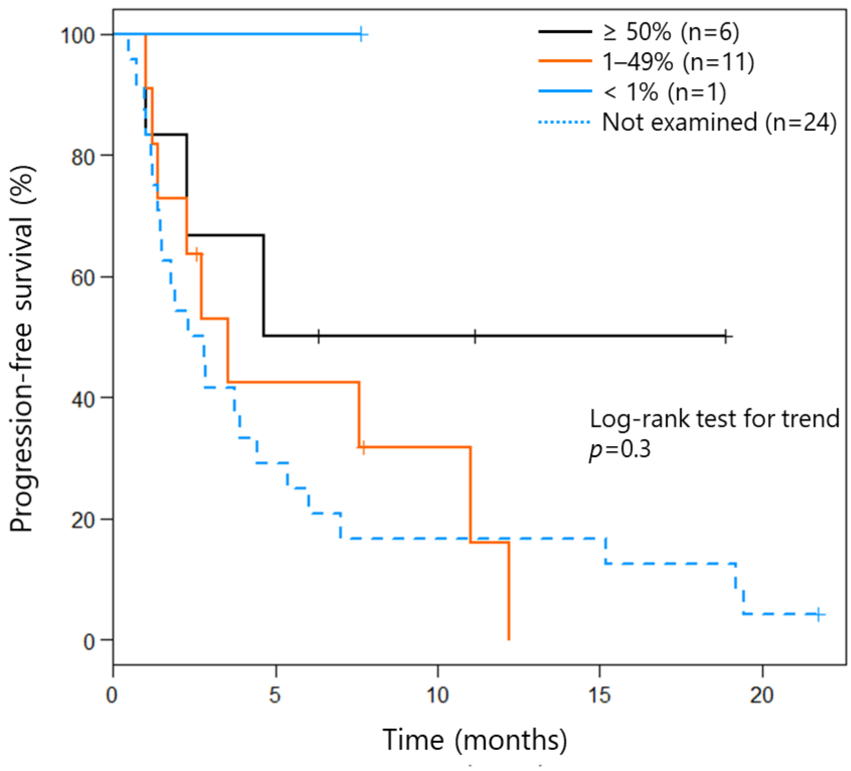

Supplement: Supplementary file 1 — Supplementary Figure S1, S2 [file 41598_2019_39120_MOESM1_ESM.pdf]
